# Early Detection with Pulse Oximetry of Hypoxemic Neonatal Conditions. Development of the IX Clinical Consensus Statement of the Ibero-American Society of Neonatology (SIBEN)

**DOI:** 10.3390/ijns4010010

**Published:** 2018-03-01

**Authors:** Augusto Sola, Sergio G. Golombek

**Affiliations:** 1 Professor of Pediatrics and Neonatology, Medical Executive Director of SIBEN, Wellington, FL 33414, USA; 2 President of SIBEN, Professor of Pediatrics and Clinical Public Health, New York Medical College, 40 Sunshine Cottage RD, Valhalla, NY 10595, USA; 3 Attending Neonatologist, Maria Fareri Children’s Hospital at Westchester Medical Center, 100 Woods Road, Valhalla, NY 10595, USA

**Keywords:** pulse oximetry, hypoxia, newborn, screening

## Abstract

This article reviews the development of the Ninth Clinical Consensus Statement by SIBEN (the Ibero-American of Neonatology) on “Early Detection with Pulse Oximetry (SpO_2_) of Hypoxemic Neonatal Conditions”. It describes the process of the consensus, and the conclusions and recommendations for screening newborns with pulse oximetry.

## 1. Introduction and Methodology

For many years, the education, training, and advances in neonatology in Spanish and Portuguese speaking countries have been inconsistent—although this is also probably true for many countries. In 2004, the Ibero-American Society of Neonatology (SIBEN) was created, with the principal objective of contributing to the improvement of the quality of life for newborn infants and their families in the Ibero-American population. SIBEN is a new society, with members from 29 different countries that focuses on neonatology facilitating education, communication, and professional advancement that contributes to the welfare and well-being of newborns and their families, in order to improve neonatal outcomes in the region. Over the past years, it has been demonstrated that the process of medical consensus could be a way of increasing professional collaboration, as well as improving uniformity in the care given to newborn infants. In 2007, SIBEN began annual meetings of a Clinical Consensus Group, where we—under the guidance of an expert or opinion leader in the topic—organized several subgroups of neonatal professionals in the Ibero-American region. Each subgroup critically reviews all the available literature in order to find the answers to several questions that had been posed to them. SIBEN’s consensus process is the first of its kind in the region. It has led to active and collaborative participation of Ibero-American neonatologists of 19 countries and has significantly improved education of all participants. At SIBEN, we believe that the critical review and summary of available clinical data as well as the recommendations made by the SIBEN consensus contribute to consistent best practice for newborn care and develops a useful foundation and valuable model to reduce the gaps in knowledge and the clinical care every newborn baby receives in in this region, thus decreasing the disparity in the care provided and improving short and long-term outcomes. Several important neonatal topics, all relevant to neonatal clinical practice, have been covered so far by SIBEN’s clinical consensus including patent ductus arteriosus (PDA), hemodynamic management, bronchopulmonary dysplasia (BPD), hematology, nutrition, persistent pulmonary hypertension of the newborn (PPHN), and hypoxic ischemic encephalopathy, which have been published in consensus statements and peer reviewed journals. This paper is a summary of SIBEN’s consensus statement on newborn screening with pulse oximetry.

## 2. Background on Screening for Congenital Heart Disease

The prevalence, epidemiology, and impact of delay in the diagnosis of CHD have been described in several publications [1,2,3,4,5,6,7,8]. Critical congenital heart defects (CCHD) affect approximately 2 out of every 1000 live births; it is estimated that about 40,000 babies are born with CCHD per year in the US, and 1.35 million worldwide, including ductus-dependent lesions. CCHD represent about 40% of deaths due to congenital malformations and the majority of deaths from cardiovascular disease occurring in the first year of life. It is known that more than 30% of CCHD deaths have been attributed to errors in diagnosis or late diagnosis [9]. For example, in UK it was estimated that 25% of congenital heart disease defects are not diagnosed until after discharge from hospital, and newborns may become seriously ill or die. It is now understood that prenatal or postnatal examination is inadequate for the early detection of these potentially lethal and treatable conditions. Delay in the diagnosis of CCHD may increase the risk of death or permanent injury in newborn babies [10,11].

In 2009, de-Wahl Granelli et al. [12] published a cohort study in which 39,821 children had oxygen saturation measured by pulse oximetry (SpO_2_) in the upper and lower extremities and demonstrated acceptable test accuracy for the detection of CCHD. Ewer et al. [13], in a similar study in 20,055 asymptomatic newborns, reported similar findings and Zhao and colleagues [14] in China studied 100,000 newborns and demonstrated the same.

In 2011, the US Advisory Committee on Heritable Disorders in Newborns and Children Advisory Committee on Hereditary Diseases [15,16,17] found that there was sufficient evidence to recommend screening with pulse oximetry [1,2,3,4,5,6,7,8,9,10,11,12,13,14,15,16,17,18,19,20,21,22,23,24,25,26,27,28,29,30,31,32,33,34,35,36,37,38,39,40,41,42,43,44,45,46,47,48,49,50,51,52,53,54,55,56,57,58,59,60,61,62,63,64]. The heart defects that can be detected early are mainly the following specific lesions: hypoplastic left heart syndrome, pulmonary atresia, tetralogy of Fallot, anomalous pulmonary venous return, transposition of large vessels, tricuspid atresia, and truncus arteriosus. Screening can also detect: interrupted aortic arch, critical aortic stenosis, aortic valve stenosis, pulmonary valve stenosis. In addition, pulse oximetry screening is useful for the early detection of other conditions with neonatal hypoxemia, such as respiratory disorders (e.g., congenital pneumonia, meconium aspiration, pneumothorax, transient tachypnea of the newborn), neonatal sepsis, and pulmonary hypertension. These findings and others were summarized in a meta-analysis and systematic review by Thangaratinam and colleagues [18].

## 3. SIBEN’s Consensus on Screening with Pulse Oximetry: An Overview

Based on the issues described above, we proceeded to organize the Ninth Clinical SIBEN Consensus on “Early Detection with Pulse Oximetry (SpO_2_) of Neonatal Hypoxemic Conditions”. The concern about the late diagnosis of CCHD led to the investigation of early detection with SpO_2_ screening. These screening programs have detected other conditions that also present with hypoxemia in addition to CCHD that would have been diagnosed later if not for the evaluation with SpO_2_ [19]. In order to make recommendations for the Ibero-American region to implement programs pulse oximetry screening, 39 neonatologists and 4 neonatal nurses from 18 Ibero-American countries were invited to participate and to collaborate. They worked for several months with an intense and collaborative methodology, and met in person at San José de Costa Rica, in September 2015, during the Annual SIBEN Conference. Professor Andrew Ewer from the UK was the leader and expert opinion for this ninth SIBEN’s Consensus. Neonatal hypoxemia, such as it occurs in critical congenital heart disease (CCHD) and other conditions, is an abnormal situation, potentially fatal if not diagnosed or if diagnosed late, PO screening allows earlier detection and thus the opportunity to optimize their management and improve outcomes.

Several questions of clinical significance were developed on the early detection with SpO_2_ of diseases that present with neonatal hypoxemia. They included:

1. Cyanosis and related concepts.
2. What is hypoxemia?
3. What is hypoxia?
4. What is pulse oximetry, and what are the normal values in a healthy term newborn?
5. What is the hemoglobin dissociation curve?
6. How does altitude influence on the SpO_2_?
7. Which are the lesions that can be detected early?
8. How should you do the screening?
9. What are normal and abnormal results?
10. What are false positive and false negative results?
11. How should you interpret the pre- and post-ductal SpO_2_ difference?
12. What should we do with an apparently healthy newborn that fails the screening?
13. How should we take care of the family of a newborn that has either a positive or negative screening?
14. Is this program cost-effective?
15. When should we order an echocardiogram?
16. Importance of the information and participation of the healthcare team—what data should you record?
17. Who should do the screening?
18. What limitations does pulse oximetry have?
19. What role can the Perfusion Index (PI) have during the screening?

The subgroups were tasked with answering 2–4 of the above questions. They methodically searched and reviewed the available literature, then interacted and worked together as a whole group to find consensus for the answers to all the questions. This SIBEN Clinical Consensus Group concluded that pulse oximetry is a non-invasive method that allows the rapid measurement of saturation of hemoglobin in arterial blood that can detect hypoxemia in asymptomatic and apparently healthy newborns who suffer from severe health conditions such as critical congenital heart disease. In addition to CCHD, the following conditions can be diagnosed early with SpO_2_ screening:

- Early sepsis
- Congenital pneumonia
- Pulmonary hypertension
- Meconium aspiration
- Transient tachypnea
- Pneumothorax
- Other various less frequent neonatal conditions

The early use of pulse oximetry in apparently healthy babies is simple, very easy to perform, fast, non-invasive, cost effective [20,21], and provides a significant improvement in quality and safety in neonatal healthcare. Thus, SIBEN recommended that programs of early detection or screening with SpO_2_ are implemented in all places where neonatal care is delivered in Latin America. In summary, the early evaluation of all the newborns with SpO_2_ is a complementary, non-invasive, easy-to-perform, and low cost test that is performed between 12–48 h of life and is of great clinical utility to detect potentially serious diseases in asymptomatic and apparently healthy newborn infants. The universal implementation of this evaluation in clinical practice leads to a narrowing of the diagnostic gap for newborns to increase patient safety and to reduce the morbidity, sequelae, and mortality of these babies.

## 4. SIBEN’s Consensus on Screening with Pulse Oximetry: A Summary

We summarize answers to some of the specific questions and recommendations below.

### 4.1. Evaluation with SpO_2_ Monitors and Sampling Sites

Neonatal screening for the detection of pathologies associated with hypoxemia has been introduced in clinical practice in the USA since 2011. Since then, studies and meta-analysis [18] show that it meets the criteria for population screening test, as well as being a tool in the early and timely diagnosis of severe neonatal conditions. Nevertheless, it is still not universally used in Latin America and work was needed to identify protocols. The SpO_2_ screening technique is very easy to perform, and should be performed in all apparently healthy newborns between 12–48 h after birth (see below) or before discharge. It should be done by placing a sensor in the palm of the right hand (pre-ductal) and then another in one of the lower limbs (post-ductal). SpO_2_ readings are taken and recorded from the two sites, one after the other (it is not necessary to use two monitors simultaneously). Screening has to be done with both pre- and post-ductal measurements because some heart defects with obstruction of the left output tract may not be diagnosed when performing a single post-ductal measurement.

The published evidence is clear in relation to the quality of the signal. The SIBEN consensus concludes that evaluating the quality of the signal is fundamental to being able to interpret that the SpO_2_ readings are correct. Therefore, the screening must be performed with a SpO_2_ monitor that functions in low perfusion states and is not subject to motion artefact.

### 4.2. Clinical Protocol

**Figure 1:**
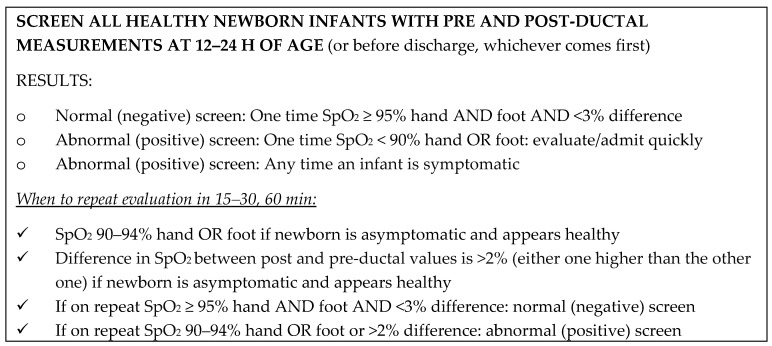
Screening protocol-algorithm recommended by SIBEN.

Based on the SIBEN clinical consensus, it is recommended that this SpO_2_ screening method (pre and post ductal) be performed in all healthy newborns between 12 and 24 h of life, or before discharge home if the discharge is prior to that age. If the first pre- and post-ductal SpO_2_ measurements are both 95–100% with <3% difference between them, the evaluation is normal and the newborn has a negative screening test. If the first measurement is positive/abnormal (SpO_2_ 90–95% and/or difference >2%) and the infant looks healthy, the pre- and post-ductal measurements must be repeated once more, according to the protocol chosen by SIBEN, described in Figure 1 below. In infants with clinical symptoms or when SpO_2_ is <90%, prompt admission to NICU and further evaluation should be initiated without delay.

A second measurement is only done if the first one is positive (abnormal) and if the infant continues to appear completely healthy. If the infant has any clinical signs, they should be admitted, as would any sick neonate for any other reason. The second evaluation should be done 15–30 min after the first in order to reduce delay. Furthermore, if the infant was sound asleep during the first evaluation, they should be alert for the second. If the second measurement is normal, the test is considered normal, that is to say that the screening is negative. If first and second evaluations are positive, and/or if the infant has any clinical signs, immediate admission to NICU is recommended. If the infant appears healthy, they should be carefully assessed as described below.

### 4.3. What to Do with a Neonate Who Appears Clinically Healthy but Has Abnormal or Positive SpO_2_?

Do not ignore the test and humbly accept that we may be wrong in our clinical assessment. It is necessary to evaluate quickly, in a detailed and complete approach, every newborn who has an abnormal test result with SpO_2_. The absence of a murmur, normal blood pressure, or the presence of normal femoral pulses do not rule out a critical congenital heart disease. In addition, in infectious conditions or other hypoxemic conditions there will be no heart murmur and the other parameters may well be normal initially. If the diagnosis is not clear, other studies should be carried out for timely diagnosis, including frequent or continuous assessment of pre- and post-ductal SpO_2_. According to the clinical suspicion, a complete diagnostic approach and may include complete blood count, cultures, blood gases, and chest X-rays. Some will require an echocardiogram, and in some it will be necessary to immediately start an infusion of prostaglandin to maintain patency of the ductus arteriosus.

### 4.4. Concept of False Positive and False Negative Screening

As mentioned, an infant with a positive screening test has one SpO_2_ < 90% or two consecutive tests with SpO_2_ 90–95% and/or pre-post ductal difference >2%. A false positive result is when the infant is found NOT to have CCHD. This occurrence is extremely rare (<0.1%) if the screening method and protocol are followed rigorously—although it may be up to 1% [22]. If the evaluation with SpO_2_ is done before 12 h of age there are slightly more false positives but diagnosis of infectious and respiratory causes of hypoxemia are more common. A false negative is when the evaluation with SpO_2_ is normal (negative screening) but the infant is actually found hours or days later to actually have CCHD. As it can be easily understood, a false negative would be a significant issue. Most studies indicate that the most frequently undiagnosed lesions are left sided obstructed lesions with obstruction to the outflow of the aorta (e.g., coarctation of the aortic arch, hypoplastic left heart, aortic stenosis) which are not necessarily associated with hypoxemia. False negatives can also occur when not using appropriate technology. The use of the preductal and postductal saturation difference and the perfusion index improve detection, but they are not infallible either.

### 4.5. Altitude and Neonatal SpO_2_ Screening

This is a topic that was addressed extensively, including physiology and alveolar gas equation. SIBEN’s consensus found that, on average, SpO_2_ values are not different in the first 12–24 h of life in infants born at less than 2500 m (about 8200 feet) above sea level. Therefore, if screening is done as mentioned and at the age recommended here, the values for positive and negative results could be kept the same. The issue is that the mean normal SpO_2_ is a bit lower at higher altitude (93–96%) but with larger standard deviations. Therefore, some totally normal babies can have SpO_2_ of 91–94% at >2700 m above sea level. So, more detailed observation would be recommended for asymptomatic infants, exercising caution and avoidance of aggressive investigations in order to prevent increasing the number of false positives. Still, exact cut-off points in moderate and high altitudes are not precisely known to adequately balance sensitivity with false positive rates. 

### 4.6. Care of the Family with an Abnormal or Positive Screening

In the first hours after birth, various events generate great emotional tension. Health care providers should make an effort to decrease this by all possible means. Families should always actively participate in the care of their newborn infant and they should also be involved during SpO_2_ screening. As this can be stressful for some parents, every effort should be made so that they clearly understand what is being done to their babies and why. Studies have shown that parents who have been well informed are mostly satisfied with the SpO_2_ screening test and have perceived the screening as valuable test to detect sick babies. In addition, parents of neonates who had a false positive result did not show greater anxiety than those with negative or normal screenings [23].

It is recommended that parents of apparently normal newborns receive written information on the SpO_2_ screening test. This written information must be accompanied by clear verbal information and clarification of any doubts that may have arisen with the information received. It is also recommended that the screening is performed with the parents present. In the face of a positive result, appropriate information and support is essential throughout.

## 5. Summary and Discussion

We have reviewed the evidence in a formal process of clinical consensus and presented the available data that demonstrates that early evaluation with pulse oximetry in apparently healthy newborns does easily detect asymptomatic newborns with severe health conditions, such as critical congenital heart disease, respiratory disorders, neonatal sepsis, persistent pulmonary hypertension, and other hypoxemic pathologies. The objective of implementing systematic protocols in clinical practice for the screening of all newborns by early pulse oximetry is to detect pathologies with early hypoxemia and to perform a therapeutic approach without delays. The consensus group of SIBEN, concludes that adequate early monitoring of SpO_2_ in apparently healthy newborns is useful for early detection of several neonatal conditions which evidence has shown that the diagnosis is sometimes untimely or late. It was estimated that about 2000 neonates died or were diagnosed late each year in the US, and that around 300,000 babies per year die worldwide because of this. The number of undiagnosed cases in developing countries is higher than in developed nations and it is estimated that less than half of the cases of CCHD are diagnosed in the first week of life. The prenatal diagnosis of CCHD can improve perinatal outcomes for certain lesions [54,55]. Recent evidence shows that CCHD detection has progressively increased from 2006 to 2012, but also that prenatal detection is highly variable in different countries [56]. In some cases, the diagnosis of fetal CCHD is made to later see that the newborn is healthy. Repeated prenatal ultrasounds are much more difficult and costly than simple SpO_2_ screening. Early diagnosis of CCHD in postnatal life significantly decreases morbidity and mortality rates [24].

The effectiveness of screening is also shown in recent publications on home births in The Netherlands [58], as well as other very comprehensive reviews [59,60,61]. Adding detailed physical examination to early evaluation with SpO_2_ increases significantly early diagnosis of hypoxemic neonatal conditions. SIBEN underscores that neonatal screening with SpO_2_ for the specific diagnosis of early CCHD does not, of course, replace prenatal detection or clinical examination but is a very useful complement. Accurate prenatal ultrasound, physical examination, and SpO_2_ screening may increase CCHD detection rates to more than 90–95%. One of SIBEN’s recommendations is that, at the beginning of this screening program each center must use a clearly defined protocol (as described previously) and at least one quality indicator e.g., performing a random evaluation every 1–2 weeks of the number of babies with screening indication (infants that should have been evaluated) and verified that the program has been met 100% of the time. If this is not the case, processes need to be improved in order to meet the objective of the evaluation and detection of all newborn infants. The quality indicators are not only for CCHD but also for early detection of respiratory or infectious conditions. Physicians should be aware that, even though the combination of early detection with pulse oximetry with other methods of evaluation reduces errors and diagnostic errors, some babies can still be discharged without proper diagnosis.

Early detection of CCHD and hypoxemic neonatal conditions not only reduces the suffering of children and families, but it can also reduce associated costs and long-term neurological compromise by not delaying admission to a specialized care unit. This is also associated with significant reductions in mortality, better surgical outcomes, less prolonged ventilation, and diminished potential developmental problems. For all of the above, actively addressing the neonatal screening of CCHD and neonatal hypoxemic conditions can achieve a significant improvement in the quality and safety of health care, as well as cost savings. In addition, and of significant importance, the screening with pulse oximetry in the newborn has been shown to detect hypoxemia in newborns with severe conditions other than CCHD—such as respiratory problems, sepsis, and persistent pulmonary hypertension.

We conclude, together with many other authors, that significant deaths and morbidity can be avoided or significantly reduced if hospitals adopt SpO_2_ screening for early and timely detection of CCHD and other hypoxemic conditions [46,57,58,59,60,61,62]. Its implementation will benefit many newborns in Latin America, where it is estimated that 60% of neonatal deaths are preventable [63,64].
